# Supplementary material for: Polyketide synthase gene clusters in the ericoid mycorrhizal fungus Oidiodendron maius: genome-wide prediction, expression profiling, and functional characterization of knockout mutants
Source: Front Plant Sci. 2026 Sep 16;17:1924430. doi: 10.3389/fpls.2026.1924430 (PMC13623903; doi:10.3389/fpls.2026.1924430)
Supplement: Supplementary file 1 [file SupplementaryFile1.zip › Supplementary Materials/Supplementary Folder 1.zip!/Oidma1_AssemblyScaffolds_Repeatmasked/knownclusterblast/cluster2/jgi.p_Oidma1_99354_mibig_hits.html]

| MIBiG Protein | Description | MIBiG Cluster | MiBiG Product | % ID | % Coverage | BLAST Score | E-value |
| --- | --- | --- | --- | --- | --- | --- | --- |
| BAJ09787.1 | dehydrogenase | BGC0000146 | Solanapyrone\_biosynthetic\_gene\_cluster | 37 | 81.4589665653 | 117 | 2.7e-26 |
| CDM31314.1 | Short-chain\_dehydrogenase/reductase\_SDR | BGC0000667 | PR\_toxin\_biosynthetic\_gene\_cluster | 32 | 40.1215805471 | 54 | 2.1e-07 |
| EHM27499.1 | putative\_short\_chain\_oxidoreductase | BGC0000235 | Kiamycin\_biosynthetic\_gene\_cluster | 31 | 42.8571428571 | 54 | 3.7e-07 |
| AAO65349.1 | putative\_ketoreductase | BGC0000236 | Kinamycin\_biosynthetic\_gene\_cluster | 40 | 26.443768997 | 50 | 5.3e-06 |
